# Readability of Online Information on the Latarjet Procedure

**DOI:** 10.7759/cureus.49184

**Published:** 2023-11-21

**Authors:** Aathir Ahmed, Sarmed Jassim, Ahmed Karkuri

**Affiliations:** 1 Orthopaedics, Royal College of Surgeons in Ireland, Dublin, IRL; 2 Surgery, Royal College of Surgeons in Ireland, Dublin, IRL; 3 Orthopaedic Surgery, Sligo University Hospital, Sligo, IRL

**Keywords:** sports surgery, health literacy, readability, latarjet procedure, orthopaedics

## Abstract

Introduction

A common complication of first-time or recurrent shoulder dislocations is bone loss at the humeral head and glenoid. Recurrent shoulder instability is often a result of bony defects in the glenoid following shoulder dislocations. In the setting of glenoid bone loss, surgical interventions are generally required to restore stability. The Latarjet procedure is a challenging operation and, due to its complexity, may be associated with operative complications. It can be difficult to explain the procedure to patients in a manner that is easily comprehensible, which may lead to confusion or being overwhelmed with information. Hence, it is important that the information available to patients is easily accessible and perceivable to allow for adequate health literacy. Health literacy is defined as the ability to make health decisions in the context of everyday life.

Methods

The search engines Google and Bing were accessed on a single day in the month of July 2023, searching the terms "Latarjet surgery" and "Latarjet procedure." For each term on both search engines, the first three pages were evaluated, resulting in a total of 114 websites for review. Out of these, 25 websites met the inclusion criteria and underwent further in-depth analysis through the online readability software, WEB FX. This software generated a Flesch Reading Ease Score (FRES) and a Reading Grade Level (RGL) for each website.

Results

In our study, the mean FRES was 50.3 (SD ±12.5), categorizing the data as 'fairly difficult to read.' The mean RGL score was 8.12 (SD ±2.35), which exceeds the recommended target.

Conclusion

In conclusion, the results of this study have demonstrated that the material available on the Internet about the Latarjet procedure is above the recommended readability levels for the majority of the population. Our findings align with similar studies assessing the readability of online patient information. Based on these findings, physicians should provide patients with vetted information to facilitate a better understanding of the procedure, thereby enabling patients to make more informed decisions regarding their health.

## Introduction

The shoulder joint is comprised of four separate articulations: sternoclavicular joint, glenohumeral joint, acromioclavicular joint, and scapulothoracic joint. The humeral head sits on the glenoid fossa, which is relatively shallow, allowing for a large range of motion in all three planes at the glenohumeral joint. However, this large range of motion inherently makes the shoulder joint the most unstable joint in the body, resulting in 50% of all joint dislocations [1]. The estimated incidence of shoulder dislocations reported is 12 per 100,000/year [2]. A common complication of first-time or recurrent shoulder dislocations is bone loss at the humeral head and glenoid [3]. Bone loss at the anteroinferior aspect of the glenoid is reported to occur in up to 22% of first-time anterior dislocations and 90% in those with recurrent glenohumeral instability [4]. First-time glenohumeral dislocations are initially managed conservatively with closed reduction and immobilization, followed by a progressive range of motion and strengthening exercises [5]. Recurrent shoulder instability is often a result of bony defects in the glenoid following shoulder dislocations. In the setting of glenoid bone loss, surgical interventions are generally required to restore stability [6,7]. In 1954, Latarjet suggested that the coracoid process could be transferred and fixed with a screw to the margin of the glenoid [8]. The proposed mechanism of restoring stability includes: (1) The use of the coracoid as a bone block augmentation, thus increasing the anteroposterior diameter of the glenoid and allowing greater bone-to-bone contact during range of motion; (2) The creation of a sling effect by the conjoint tendon and lower subscapularis to reinforce the anterior joint capsule, especially when the arm is in an overhead position; and (3) The repair of the anterior joint capsule using the coracoacromial ligament [9].

The Latarjet procedure is a challenging operation and, due to its complexity, may be associated with operative complications [10]. It can be difficult to explain the procedure to patients in a manner that is easily comprehendible, which may lead to confusion or being overwhelmed with information. These patients will often turn to the Internet to help them gain a better understanding of their procedure and specific postoperative activities and recovery [11]. Hence, it is important that the information available for patients is easily accessible and perceivable to allow adequate health literacy. Health literacy is defined as the ability to make health decisions in the context of everyday life. It is an issue that many patients regularly encounter, despite being literate in other areas. This challenge often arises due to the use of unfamiliar medical vocabulary and the complex healthcare settings they find themselves in [12]. Lower levels of health literacy are associated with higher rates of complications and rehospitalization following discharge [13]. Health literacy is an independent and modifiable risk factor that can reduce the incidence of postoperative complications. A few simple steps to improve health literacy include using simple language, incorporating graphs and pictures instead of long written instructions, and providing information at an appropriate grade level [14]. The average reading grade level for the American population is at the 7th to 8th grade level [15]. Taking this into account, a complex procedure such as the Latarjet, with its extensive period of rehabilitation [16], can lead to reduced compliance and affect postoperative complication rates and patient satisfaction if the information is not fully understood. The U.S. Department of Health and Human Services has proposed that patient information documents be written at a reading grade level (RGL) of no higher than the sixth grade to help increase health literacy [17]. However, previous studies have shown that most online patient information is at a readability level that exceeds the recommended level [18-20]. Following an extensive literature review of the MEDLINE database, we have not found a previously published paper describing the access to information on the Latarjet procedure. The aim of this study is to analyze the readability of digital information regarding the Latarjet procedure.

## Materials and methods

The search engines Google and Bing were accessed on a single day in July 2023, searching for the terms “Latarjet surgery” and “Latarjet procedure”. For both terms across each search engine, the first three pages were evaluated, resulting in a total of 114 websites for review. Out of these, 25 websites met the inclusion criteria and underwent further in-depth analysis using the online readability software, WEB FX. The search was limited to the first three pages, as prior studies have demonstrated that the majority of the public do not search past the second page, with most limiting their search to only the first page of a search engine [21,22]. The results for each search term and search engine are detailed in Table 1.

**Table 1:** Results of search terms.

Search Engine	Search Term	Number of Results
Google	Latarjet Procedure	982,000
Google	Latarjet Surgery	1,440,000
Bing	Latarjet Procedure	3,680,000
Bing	Latarjet Surgery	61,300

**Figure 1:**
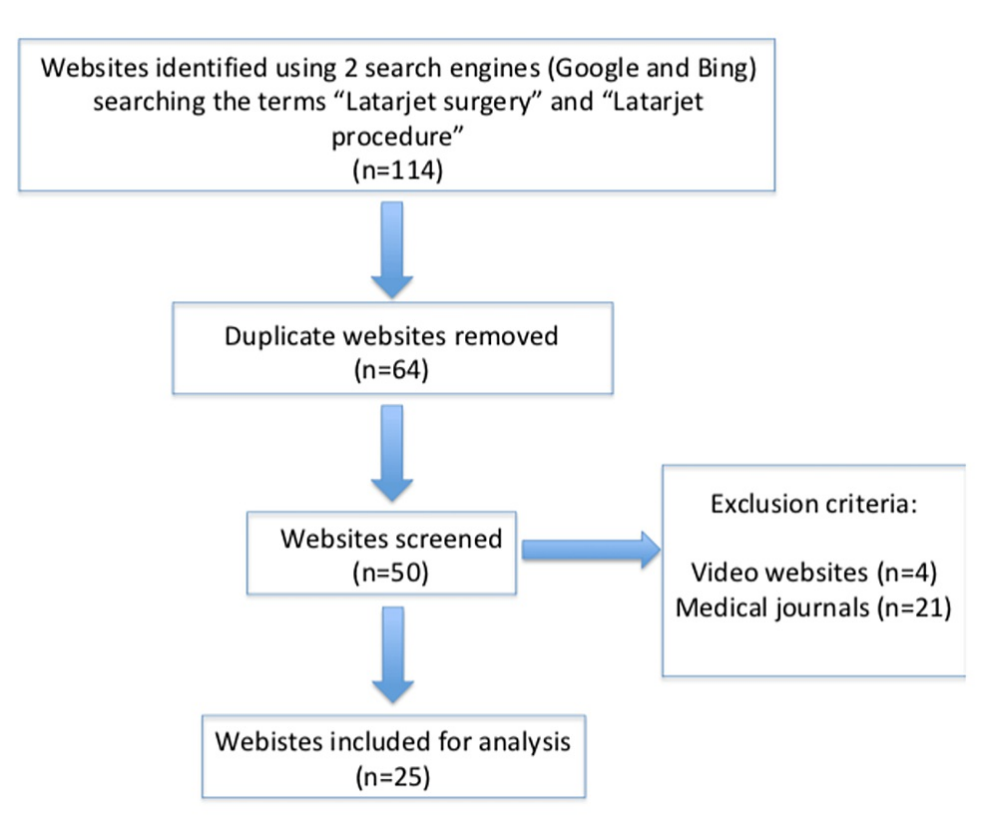
Flowchart of inclusion methodology.

Once duplicate websites were removed, the remaining ones were evaluated based on the exclusion criteria. These criteria included websites requiring logins, medical journals, and websites primarily comprised of video content. Following these exclusions, we were left with 25 websites that met the inclusion criteria and were selected for further in-depth analysis. A flow chart illustrating this methodology is presented in Figure 1.

The 25 websites were then classified into categories, including physician, non-physician, academic, commercial, media and news, social media, and non-specified. Academic websites were those affiliated with a University or teaching hospital. Physician websites referred to private websites run by doctors in private practice, while non-physician websites were linked with multidisciplinary team members such as physiotherapists. Commercial websites were associated with the marketing of a product. A list of all included websites is referenced for review in Table 2.

**Table 2:** List of websites included for analysis.

Websites
http://thelondonshoulderpartnership.co.uk/shoulder/shoulder-surgery/latarjet-anterior-stabilisation/
http://www.orthosports.com.au/pdf-download/Latarjet%20procedure.pdf
https://drrossradic.com.au/shoulder/shoulder-procedures/latarjet-procedure/
https://en.wikipedia.org/wiki/Latarjet_procedure
https://lasportsorthomd.com/latarjet-procedure-surgery-van-nuys-thousand-oaks-ca/
https://matthewprovenchermd.com/open-shoulder-stabilization-latarjet-procedure-vail-aspen-denver-co/
https://radiopaedia.org/articles/latarjet-procedure
https://sydneyshoulderunit.com.au/procedures/latarjet-procedure/
https://www.arthrex.com/shoulder/latarjet-coracoid-process-transfer-for-glenoid-deficiency
https://www.brighamandwomens.org/assets/BWH/patients-and-families/rehabilitation-services/pdfs/shoulder-latarjet.pdf
https://www.domontortho.com/latarjet-procedure-orthopedic-surgeon-lincolnshire-libertyville-il.html
https://www.dr-meyer-orthopaedics.com/operations/shoulder/unstable-shoulder/coracoid-block/
https://www.feelgoodlife.com/latarjet-procedure
https://www.kennethcutbush.com/shoulders/shoulder-surgery/arthroscopic-latarjet
https://www.massgeneral.org/assets/mgh/pdf/orthopaedics/sports-medicine/dr-price/latarjet.pdf
https://www.melbourneshoulderandknee.com.au/shoulder-knee-surgeon-melbourne-latarjet-procedure.html
https://www.onehealthcare.co.uk/treatments/shoulder-latarjet-surgery/
https://www.orthovirginia.com/latarjet-shoulder-reconstruction/
https://www.physio-pedia.com/Latarjet_Procedure
https://www.shoulderandkneesurgery.com.au/shoulder-surgery/latarjet-procedure-shoulder-instability-perth/
https://www.shoulderdoc.co.uk/article/1543
https://www.shoulderdoc.co.uk/section/914
https://www.verywellhealth.com/latarjet-surgery-for-the-shoulder-2549892
https://www.windsorupperlimb.com/procedures/shoulder-procedures/open-stabilisation-latarjet-procedure
https://www.ypo.education/orthopaedics/shoulder/latarjet-procedure-t238/video/

Once the websites were classified into their specific categories, they were then run through the online readability software, WEB FX [23]. This software was able to generate a Flesch Reading Ease Score (FRES) and an RGL for each website. The FRES is based on a 0-100 scale. This score is generated based on an algorithm that accounts for the number of syllables and sentence length in a given article. It also considers the number of complex words used in a sentence, defined as words with three or more syllables or words with more than six letters. A high score means the text is easier to read, while low scores suggest the text is complicated to understand. A value between 60 and 80 should be easy for a 12 to 15-year-old to understand. A breakdown of the FRES scoring system is shown in Table 3 [24].

**Table 3:** Flesch Reading Ease Score (FRES). Source: [24]

Score	School Level (US)	Notes
100-90	5^th^ Grade	Very easy to read. Easily understood by an average 11 year old student
90-80	6^th^ Grade	Easy to read. Conversational English for consumers
80-70	7^th^ Grade	Fairly easy to read
70-60	8^th^ & 9^th^ Grade	Plain English. Easily understood by 13 to 15 year old students
60-50	10^th^ to 12^th^ Grade	Fairly difficult to read
50-30	College	Difficult to read
30-10	College Graduate	Very difficult to read. Best understood by university graduates
10-0	Professional	Extremely difficult to read. Best understood by university graduates

The RGL accounts for how easy it is to read and understand a text on the first pass. It is based on the American schooling system and represents the cumulative years of schooling required to easily comprehend the given text. As demonstrated previously, it has been recommended that patient information material should not be written at a level greater than the 6th grade to maximize readability [18-20].

## Results

From the initial 114 websites identified, 25 websites were included for evaluation using the online readability software, WebFX. This included 13 physician websites, three non-physician websites, four non-specified websites, three media and news websites, one academic website, and one commercial website. The mean FRES score was 50.3 (SD +/- 12.5), which categorizes the data as "fairly difficult to read" (Table 3). Seventeen (68%) of the websites had a FRES score less than 50, indicating that they would require the reader to be at the college level or higher to read and understand most of the material available online. The commercial website had the lowest mean FRES score, indicating that it was the most difficult to read. Surprisingly, the academic website had the highest mean FRES score, giving it an "easy to read" ranking. This is outlined in Table 4.

**Table 4:** Breakdown of FRES and RGL scores based on website category. FRES: Flesch Reading Ease Score; RGL: Reading Grade Level.

	Type of website	N	Mean	Median	SD	Minimum	Maximum
FRES	Physician	13	45.25	45.9	6.41	31.7	57.5
	Non-physician	3	68.4	78.6	23.707	41.3	85.3
	Non-specified	4	48.23	44.8	7.839	43.4	59.9
	Media and news	3	52.03	52.2	4.053	47.9	56
	Commercial	1	42.5	42.5	NaN	42.5	42.5
	Academic	1	73.5	73.5	NaN	73.5	73.5
RGL	Physician	13	9.19	8.7	1.622	7.5	13.2
	Non-physician	3	4.73	3.1	3.636	2.2	8.9
	Non-specified	4	7.92	7.9	0.737	7.1	8.8
	Media and news	3	8.2	8.5	0.985	7.1	9
	Commercial	1	9.3	9.3	NaN	9.3	9.3
	Academic	1	3.7	3.7	NaN	3.7	3.7

**Figure 2:**
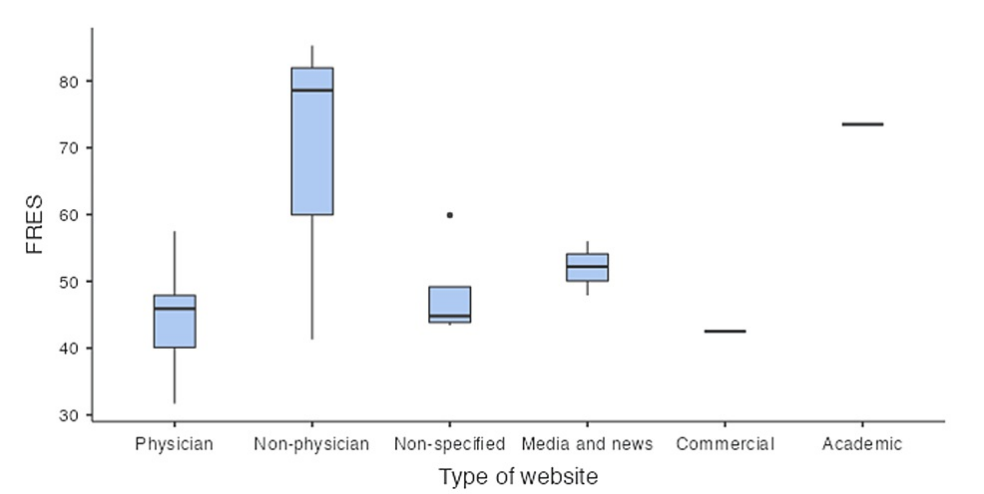
Box plot of Flesch reading ease score. FRES: Flesch Reading Ease Score.

**Figure 3:**
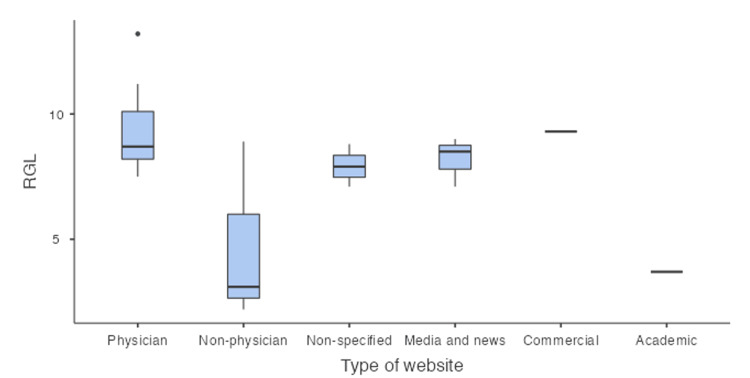
Box plot of reading grade level. RGL: Reading Grade Level.

The mean RGL score was 8.12 (SD +/- 2.35), which is higher than the recommended target. Seventeen (68%) of the websites had an RGL of 8 and higher, again demonstrating that most of the material is written above the recommended level for readability. Table 4 demonstrates that the commercial website had the highest mean RGL, while the academic website had the lowest mean RGL, again making the academic website the easiest to read. Both the FRES score and RGL score demonstrate that most websites are written at a level above the recommended target. Box plot representations of the FRES and RGL scores are illustrated in Figures 2-3, respectively.

## Discussion

The Latarjet procedure is the gold standard treatment for recurrent shoulder instability associated with bone loss [25]. Despite excellent reported outcomes, it is a technically and biomechanically complex operation with an intensive postoperative rehabilitation protocol. Non-compliance with postoperative instructions carries a high risk of complications [25]. Thus, patients require access to easily comprehensible information to make informed decisions. In recent years, the Internet has changed the way patients access information regarding various medical conditions, thus allowing them to carry out their own research for a "second opinion." However, there is very little regulation of the information that is available online, which may result in misinformation being distributed. A potential consequence of this misinformation is cyberchondria, which is defined as the mental state of stress and anxiety evoked by excessive research of information on the Internet [26]. Unintentionally, as patients believe inaccurate information on the Internet to be the truth, it can result in difficult pre-operative consultations to iron out misconceptions about a certain procedure or medical treatment. This, in turn, can negatively impact doctor-patient relationships and result in patient harm if misinformed decisions are made [27]. Therefore, it is essential that the information available online is easily accessible, accurate, and, above all, expressed at a level that is comprehensible to the general population. The verified readability tool that was used in our study was the FRES system, which incorporates the average sentence length and average syllables per word into a formula. This allows the author to optimize readability using shorter sentences and words [28]. In our study, the mean FRES score was 50.3, indicating that the material was difficult to read overall. The main finding in our study was that the information available on the Internet is written at a level that is too difficult to read and, therefore, understand for the general population. The majority of the websites providing information on the Latarjet procedure were from the physician category, followed by the non-physician category. Our results were in keeping with similar studies performed assessing the readability of online patient information [18,20,21,22].

The second assessment of readability was carried out using the RGL. Different sources have recommended different RGLs for online medical information. The consensus is that an RGL between the 6th and 8th grades is acceptable [29,30]. Our study found a mean RGL of 8.12, with 68% being over the 8th-grade level and only 12% below the 6th-grade level. Surprisingly, the academic websites in our study had the lowest mean RGL of 3.70 compared to the overall mean RGL. This may imply that academic institutions are making a conscious effort to balance out the longstanding issues of high readability levels online. There were a few limitations in our study that we have acknowledged. Firstly, the search for terms was carried out at a single point in time. This only provided us with a cross-sectional snapshot of the information. Secondly, the data represented may be skewed in the direction of representing national websites, as the search was conducted in a single country (Republic of Ireland). Thirdly, while the two most common search engines were used, we did not account for other platforms from which patients may source their information.

## Conclusions

In conclusion, the results of this study have demonstrated that the material available for patients on the Internet regarding the Latarjet procedure is above the recommended readability levels for most of the population. Our results are in keeping with similar studies performed assessing the readability of online patient information. Based on these findings, physicians should provide information to their patients that they have vetted to allow them to better understand the procedure and, therefore, make a more informed decision regarding their health.
